# LnNP@ZIF8 Smart System for In Situ NIR-II Ratiometric Imaging-Based Tumor Drug Resistance Evaluation

**DOI:** 10.3390/nano12244478

**Published:** 2022-12-17

**Authors:** Qingyuan Wang, Zhizheng Zhang, Dehui Qiu, Xuanxiang Mao, Zhaoxi Zhou, Tiansong Xia, Jifu Wei, Qiang Ding, Xiaobo Zhang

**Affiliations:** 1 Department of Breast Surgery, The First Affiliated Hospital of Nanjing Medical University, 300 Guangzhou Road, Nanjing 210029, China; 2 State Key Laboratory of Analytical Chemistry for Life Science, School of Chemistry and Chemical Engineering, Nanjing University, Nanjing 210023, China; 3 Department of Pharmacy, Jiangsu Cancer Hospital, The Affiliated Cancer Hospital of Nanjing Medical University, Jiangsu Institute of Cancer Research, Nanjing 210009, China; 4 Department of Clinical Pharmacy, School of Pharmacy, Nanjing Medical University, Nanjing 211103, China

**Keywords:** lanthanide doped nanoparticle, ZIF8, NIR-II ratiometric imaging, smart drug delivery system, tumor drug resistance evaluation

## Abstract

Just-in-time evaluation of drug resistance in situ will greatly facilitate the achievement of precision cancer therapy. The rapid elevation of reactive oxygen species (ROS) is the key to chemotherapy. Hence, suppressed ROS production is an important marker for chemotherapy drug resistance. Herein, a NIR-II emission smart nanoprobe (LnNP@ZIF8, consisting of a lanthanide-doped nanoparticle (LnNP) core and metal-organic framework shell (ZIF8)) is constructed for drug delivery and in vivo NIR-II ratiometric imaging of ROS for tumor drug resistance evaluation. The drug-loaded nanoprobes release therapeutic substances for chemotherapy in the acidic tumor tissue. As the level of ROS increases, the LnNPs shows responsively descending fluorescence intensity at 1550 nm excited by 980 nm (F1550, 980Ex), while the fluorescence of the LnNPs at 1060 nm excited by 808 nm (F1060, 808Ex) is stable. Due to the ratiometric F1550, 980Ex/F1060, 808Ex value exhibiting a linear relationship with ROS concentration, NIR-II imaging results of ROS change based on this ratio can be an important basis for determining tumor drug resistance. As the chemotherapy and resistance evaluation are explored continuously in situ, the ratiometric imaging identifies drug resistance successfully within 24 h, which can greatly improve the timeliness of accurate treatment.

## 1. Introduction

Despite tremendous advances in targeted therapy and immunotherapy [1,2,3], cytotoxic chemotherapies remain the backbone for cancer treatment at different stages [4,5]. The commonly used chemotherapeutic drugs for breast cancer include doxorubicin (DOX), paclitaxel, and cyclophosphamide [6,7]. Studies have shown that one of the main mechanisms of doxorubicin in tumor treatment is to stimulate an intracellular oxidative stress state to produce excessive intracellular reactive oxygen species (ROS) levels, causing death signals such as DNA damage and activation of apoptosis [8]. However, to compensate for this and maintain redox homeostasis, tumor cells can develop a set of complex resistant mechanisms during treatment, such as enhancing antioxidant capacity [9,10]. Recently, more attention has been focused on the antioxidant system in tumors [11,12]. Unfortunately, there is no technology available for detecting in situ drug resistance to chemotherapy. Hence, the ability to relieve oxidative stress and elevated antioxidant capacity are potential hallmarks of drug-resistant cancer [13]. Based on this principle, in situ ROS monitoring during chemotherapy will be used as a basis for the evaluation of tumor drug resistance.

**Figure 1:**
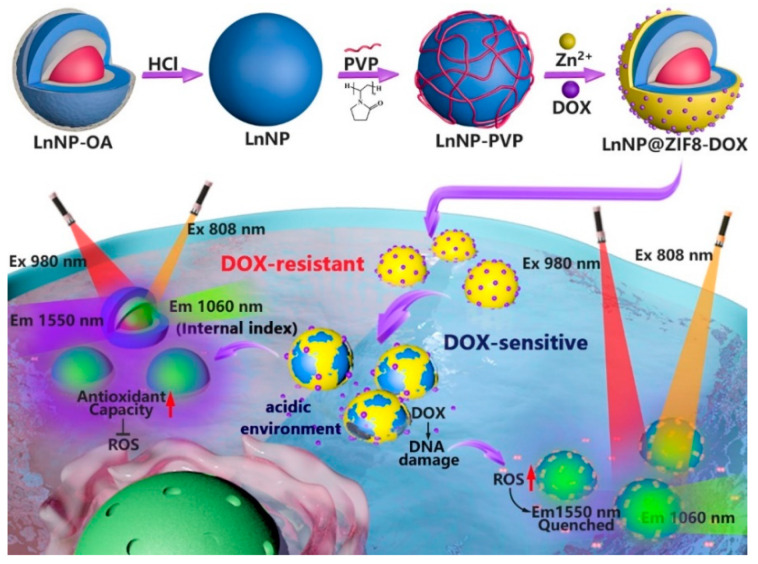
Schematic diagram of NIR-II-based in situ ratiometric tumor resistance evaluation.

For this, a NIR-II (wavelengths > 1000 nm) emission smart nanoprobe (LnNP@ZIF8, consisting of a lanthanide-doped nanoparticle (LnNP) core and metal-organic framework shell (ZIF8)) was constructed for drug delivery and NIR-II ratiometric imaging of ROS for tumor drug resistance evaluation in vivo (Figure 1). NIR-II-based imaging provides more accurate in situ information due to the high tissue penetration of NIR-II and the avoidance of autofluorescence and scattering of conventional fluorescence in the visible region [14,15,16]. In this work, multi-layered core-shell structured LnNPs were the NIR-II signal source for drug resistance evaluation [17,18], and the metal-organic frameworks (MOF) ZIF8 acted as a smart porter to release chemotherapeutic drugs in an acidic environment [19,20,21]. As a proof of concept, DOX, the most commonly used chemotherapeutic drug, was explored in this study [22]. ROS are defined as unstable molecules that contain oxygen. Hydrogen peroxide (H_2_O_2_) is one of the most common ROS species [23]. Therefore, we first successfully performed in vitro validation of the ROS response using H_2_O_2_.

Once the DOX-loaded probe was captured by cancer cells, the outer ZIF8 would gradually degrade under acidic conditions (pH ≈ 5.5) and release the drug (Figure 1). Subsequently, the exposed LnNPs would be monitored for in situ ROS levels. To avoid signal fluctuations due to differences in probe uptake, the stable emission of 1060 nm light from the LnNP core at 808 nm excitation (F1060, 808Ex) can be used as an internal index for signal correction. At the same time, the outer layer of the LnNP core emits intense 1550 nm light (980 nm excitation) (F1550, 980Ex) at low levels of ROS. However, this phenomenon indicates that ROS production had been inhibited and was in a drug-resistant state (Figure 1, lower left pink area). In contrast, there was a rapid rise in ROS in drug-sensitive cells and a trend toward quenching of F1550, 980Ex (Figure 1, lower right blue area). The final ratio of F1550, 980Ex to F1060, 808Ex is a quantitative assessment of in situ tumor drug resistance.

To our knowledge, this is the first work to report a spatiotemporal real-time ratiometric infrared monitoring evaluation of tumor ROS levels in vivo during chemotherapy. This redox imaging probe has the potential to inform therapeutic response and drug resistance. Ultimately, this would facilitate early intervention, allowing the clinician to adapt the treatment regimen, with the potential to achieve a better prognosis.

## 2. Materials and Methods

### 2.1. Materials and Apparatus

Anhydrous yttrium chloride (YCl_3_) (99.9%), anhydrous gadolinium chloride (GdCl_3_) (99.9%), anhydrous ytterbium chloride (YbCl_3_) (99.9%), anhydrous neodymium trichloride (NdCl_3_) (99.9%), anhydrous erbium chloride (ErCl_3_) (99.9%), anhydrous cerium chloride(CeCl_3_) (99.9%), cyclohexane, chloroform, hydrogen peroxide (H_2_O_2_), hydrochloric acid (HCl), sodium hydroxide, ammonium fluoride (NH_4_F), cyclohexene, oleic acid (OA), 1octadecene (ODE), and polyvinyl pyrrolidone (PVP) were purchased from Aladdin Industrial Co. (Shanghai, China). Female Balb/c mice and severe combined immunodeficiency (SCID) mice were purchased from the Model Animal Research Center of Nanjing University (MARC, Nanjing, China).

Transmission electron microscopic (TEM) images were captured on a JEM-2100 transmission electron microscope (JEOL Ltd., Tokyo, Japan). Formvar/Thick Carbon Film Coated grids (BZ11024a) were purchased from Beijing zhongjingkeyi Technology Co. (Beijing, China). and the accelerating voltage was 200 kV. Dynamic light scattering (DLS) was conducted on a ZetaPlus 90 Plus/BI-MAS particle size analyzer (Brook haven, Atlanta, GA, USA). The crystal structures of the obtained materials were characterized by X-ray diffraction (XRD) performed on an Ultima IV 285 X-ray powder diffractometer (Rigaku Co., Tokyo, Japan). NIR-II images were captured by an IVIS Lumina XR III in vivo imaging system (PerkinElmer, Boston, MA, USA).

### 2.2. Preparation of NaYF_4_:Nd,Gd@NaYF_4_:Gd@NaYF_4_:Gd,Yb,Er@ NaYF_4_:Ce LnNP-OA

Synthesis of multilayer structured LnNP-OA: UCNP-OA was synthesized through seed-mediated growth. The LnNP core was firstly synthesized by adding 1 mmol of Ln(OA)_3_ (Y:Nd:Gd = 0.9:0.05:0.05) to the mixture of OA and 1-octadecene (20 mL, v:v = 1:3). After being degassed in vacuum at 150 °C for 1 h, this mixture was cooled down to 45 °C, and added with 10 mL methanol solution of NH_4_F (4.0 mmol) and NaOH (2.5 mmol) dropwise under stirring for 30 min. After the reaction mixture was heated to 110 °C to completely remove methanol, and 300 °C under a nitrogen atmosphere for 90 min, LnNPs (NaYF_4_:Nd,Gd) with a diameter of 18 nm were obtained.

Various amounts of outer layer precursor were injected into the above reaction mixture of the UCNP core and stirred for 30 min to grow the emitting layer on the core (NaYF_4_:Nd,Gd@NaYF_4_:Gd). The third layer was then grown by injecting its precursor in the mixture and stirring for 30 min to get multilayer structured UCNP (NaYF_4_:Nd,Gd@NaYF_4_:Gd@NaYF_4_:Gd,Yb,Er). The four-layer structure of NaYF_4_:Nd,Gd@NaYF_4_:Gd@NaYF_4_:Gd,Yb,Er@NaYF_4_:Ce LnNP-OA can be obtained in the same way. The mixture was then cooled down to room temperature, precipitated with acetone, repeatedly washed with cyclohexane, and re-dispersed in 10 mL CHCl_3_ or cyclohexane for further use.

Composition of the LnNP Core: Y: Nd: Gd = 0.9:0.05:0.05; Shell 1: Y:Gd = 0.95:0.05; Shell 2: Y:Gd:Yb:Er = 0.39:0.39:0.2:0.02; Shell 3: Ce:Y = 0.2:0.8.

### 2.3. Preparation of PVP-Capped LnNP (LnNP-PVP)

To create a suitable surface for growing the ZIF8, OA ligands on the surface of the LnNPs were removed and then coated with a PVP layer. Briefly, LnNPs (0.4 mmol) were dispersed in 10 mL (0.1 M) of dilute HCl solution and sonicated for 1 h. Then they were washed three times with distilled water and dispersed in 5 mL of ethanol. Then, 15 mL of chloroform containing 0.96 g of PVP (Mw = 24,000) was dropwise added into the solution with vigorous stirring. After stirring for 24 h, PVP-capped LnNPs were obtained. Finally, the products were collected from the solution by centrifugation and washed with ethanol to remove excess PVP molecules.

### 2.4. Synthesis of the LnNP@ZIF8 Nanocomposites

Typically, 0.4 mL of PVP-LnNP in methanol (0.1 M) was added into 5 mL of 2-methylimidzole (0.13 g/mL) methanol solution, and stirred for about 30 min, then mixed with 5 mL of Zn(NO_3_)_2_·6H_2_O (0.059 g/mL) methanol solution. The mixture was stirred at room temperature for 24 h. The resulting white solid was collected by centrifugation, washed with methanol three times, and re-dispersed in methanol for further use.

### 2.5. Preparation of LnNP@ZIF8-DOX Nanoprobes

The dried precipitate of LnNP@ZIF8 (4 mM) was mixed with 0.5 mg, 1 mg, and 2 mg of DOX into 2 mL of distilled water for ultrasonic dispersion, respectively. The mixture was vibrated for 24 h, allowing the DOX to be adsorbed into the pores of LnNP@ZIF8. The LnNP@ZIF8-DOX nanoprobes were then obtained by centrifugation at 10,000 r/min (4480× *g*) for 5 min and washed with water three times.

### 2.6. Quantitative Detection of ROS

The LnNP@ZIF8-DOX was reacted with H_2_O_2_ with concentrations from 0 to 80 μM, and the emission spectra excited by 808 nm and 980 nm laser light were measured respectively. The fluorescence intensity ratio at 1060 nm and 1550 nm (F1550,980Ex/F1060,808Ex) was calculated. Then, 500 μL of the above mixture solution was added to a 24-well plate, and images were taken by the NIR-II fluorescence imaging setup.

### 2.7. Cell Culture

Human breast cancer DOX resistance cell line MCF-7-ADR was kindly provided by Dr. Shui Wang (Nanjing Medical University, Nanjing, China); 4T1 and MCF-7 cell lines were purchased from the American Type Culture Collection (ATCC, USA). These three cell lines were cultured in DMEM (Wisent, Nanjing, China) containing 10% FBS (Gibco, CA, USA), 4.5 mg/mL glucose, 100 μg/mL streptomycin, and 100 μg/mL penicillin (Hyclone, Logan, UT, USA), in a humidified incubator containing 5% CO2 at 37 °C.

### 2.8. In Vitro Selectivity Studies of LnNP

LnNPs (0.5 mg·ml^−1^) in PBS solution were reacted with other ROS (80 μM) and various amino acids and ions (GSH, Gly, Glu, glucose, CaCl_2_) (1 mM) for 30 min, and the emission spectra excited by 808 nm and 980 nm laser light were measured, respectively. The F1550,980Ex/F1060,808Ex ratio was calculated and a student two-sided *t*-test was performed.

### 2.9. Cell Uptake and Imaging

MCF-7 cells and 4T1 (5 × 10^5^ mL^−1^) were seeded into 12-well plates. After 8 h, these were treated with LnNP@ZIF8 or LnNP@ZIF8-DOX (100 μg/mL) and incubated for 6 h, then washed with PBS. The cells were then fixed with 1 mL of 4% paraformaldehyde for 10 min and washed with PBS. All cell samples were photographed under a NIR microscope. The NIR-II images were taken by a NIRVana HS camera (Teledyne Princeton Instruments, Trenton, NJ, USA) and processed by Lightfield software.

### 2.10. Cytotoxicity Assay

MCF-7 cells were inoculated in 96-well plates (5 × 10^3^ cells/well) and were divided into three groups: an untreated group (PBS), and groups treated with LnNP@ZIF8 (LnNP@ZIF8) or LnNP@ZIF8-DOX (LnNP@ZIF8-DOX) for 24 h. The cell survival rate was further tested by CCK-8 kit (Dojindo, Tokyo, Japan) following the manufacturer’s guidelines. After 12 h, cells were inoculated in 96-well plates (5 × 10^3^ cells/well) and grown in complete medium containing different concentrations of DOX (MedChemExpress, Monmouth Junction, NJ, USA),

### 2.11. Mice and Tumor Models

#### 2.11.1. Construction of 4T1 Orthotopic Mouse Model

Ten six-to-eight-week-old female Balb/c mice were purchased from the Model Animal Research Center of Nanjing University (MARC, Nanjing, China) and maintained under specific pathogen-free conditions in the Animal Core Facility of Nanjing Medical University. The mice were anesthetized using inhaled isoflurane (RWD Life Science, Shenzhen, China). In total, 1 × 10^5^ 4T1 cells in 100 μL of PBS were injected subcutaneously into the right inguinal mammary fat pads of each animal.

#### 2.11.2. Construction of MCF-7 Human Breast Tissue-Derived Orthotopic Mouse Model

Ten five-to-seven-week-old female severe combined immunodeficiency (SCID) mice were purchased from the Model Animal Research Center of Nanjing University (MARC, Nanjing, China). The SCID mice were kept under specific pathogen-free (SPF), temperature-controlled conditions. Cages, bedding, and drinking water were autoclaved and changed regularly. The mice were maintained in a daily cycle of 12 h periods of light and darkness. Normal human breast tissues were obtained from freshly discarded material of elective reduction mammoplasty surgery according to the ethical guidelines of the Declaration of Helsinki and approved by the ethics and research committee of the First Affiliated Hospital of Nanjing Medical University. The breast tissues were stripped of excess fat and sliced under sterile conditions into pieces of approximately 4 × 4 × 4 mm. Three pieces were selected randomly for histological examination and other pieces were placed in phosphate-buffered saline (PBS) at 0 °C until implanted in the SCID mice. Before implantation, mice were anesthetized using inhaled isoflurane (RWD Life Science, Shenzhen, China). Surgical procedures were modified according to the previous description. Briefly, 5–6 mm incisions were made by a scalpel in the skins of the left and right mid-dorsal flanks. Implantation was finished within 6 h of the mammoplasty surgery. The mice received gentamicin in their drinking water (800,000 U/L) up to 1 week following the implantation. Ten SCID tissue-derived orthotopic mice were divided into the MCF-7 WT group and the MCF-7 ADR group randomly and equally. Cells were harvested with 0.25% trypsin and 0.02% disodium edetate, washed in the medium, counted, and resuspended in PBS. One week after the implantation of human breast tissues, the MCF-7 WT cells and MCF-7 ADR cells (5 × 10^5^) in 0.2 mL PBS were inoculated into the left implanted breast tissues.

### 2.12. Mouse Treatment Procedures

Subcutaneous injection of murine 4T1 cells into Balb/c mice led to the formation of primary tumors. At 21 days after 4T1 cell injection, when the tumor diameter was >7 mm (range, 7–10 mm), ten mice were divided into two groups: treated with LnNP@ZIF8 (LnNP@ZIF8) or treated with LnNP@ZIF8-DOX (LnNP@ZIF8-DOX). The mice were anesthetized using inhaled isoflurane (RWD Life Science, Shenzhen, China). The tumor site was sanitized with a 75% ethanol tincture before treatment. All the procedures were performed aseptically. Then, a 100µL mixture of the LnNPs in PBS (5 mg/mL) was injected subcutaneously into the 4T1 tumors.

Eight of ten SCID tissue-derived orthotopic mice models constructed successfully. Then, a 100 µL mixture of the LnNP@ZIF8-DOX (5 mg/mL) in PBS was injected subcutaneously into the MCF-7 WT and MCF-7 ADR tumors.

NIR-II fluorescent images were recorded at 24 h after probe injection using an in vivo imaging system equipped with an IVIS Lumina XR III in vivo imaging system (PerkinElmer, Waltham, MA, USA).

All mice were sacrificed by cervical dislocation after CO_2_ euthanasia.

### 2.13. Declarations

All mice studies were conducted according to the Guide for the Care and Use of Laboratory Animals and approved by the Animal Care and Use Committee of Nanjing Medical University. The ethical review number was No.2022-SR-454. All experimental methods were in accord with the Helsinki Declaration.

### 2.14. Statistical Analysis

Origin 2018 64 Bit and GraphPad Prism (Version 8.0) were used to analyze any differences present between groups in the data. Significance was analyzed by the Student *t*-test. All experiments were independently repeated three times, and *p* < 0.05 was used for statistical significance.

## 3. Results and Discussion

### 3.1. Preparation and Characterization

**Figure 2:**
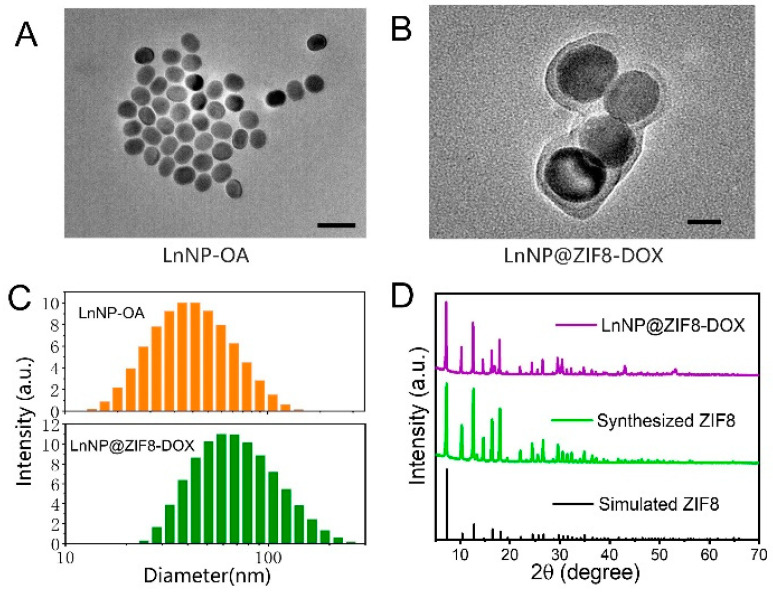
(**A**) Transmission electron microscopy (TEM) image of LnNP-OA (CS1S2S3) (Scale bar: 80 nm) and (**B**) LnNP@ZIF8-DOX nano-assemblies (Scale bar: 25 nm). (**C**) Distribution of DLS for LnNP-OA and LnNP@ZIF8. (**D**) XRD patterns of LnNP@ZIF8-DOX nano-assemblies, synthesized ZIF8, and simulated ZIF8.

We designed lanthanide-doped nanoparticles (LnNPs) with multilayer core shells (Figure 2A, 44.1 ± 2.1 nm) with NIR orthogonal excitation characteristics. Firstly, hydrophobic NaYF_4_:Nd,Gd nanoparticles were synthesized as the core of the nanoprobe via a facile high-temperature co-precipitation method [24,25]. The core is wrapped in inert Shell 1 (S1) and emits 1060 nm light stably under 808 nm excitation, which acts as an internal standard during the detection process (Figure S1). Shell 2–3 forms a sensor responsive to ROS, with its 1550 nm emission decreasing as ROS rises under 980 nm excitation (Figure S2). To create a suitable surface for growing the ZIF8, hydrochloric acid was used to remove the OA ligands. Then, polyvinylpyrrolidone (PVP) was coated onto the bare LnNPs. After stirring for 24 h, PVP-capped LnNPs were obtained. Finally, a ZIF8 layer mixing DOX aqueous solution was coated onto the LnNP nanodisks to form the hydrophilic LnNP@ZIF8-DOX core−shell nanostructure. Transmission electron microscopy (TEM) images clearly showed the obvious core-shell geometry (57.5 ± 4.1 nm) (Figure 2B), indicating the successful synthesis of LnNP-OA structures (Figure S3 and Figure 2A). The DLS data likewise confirm this parcel trend (average diameter increased by 16.2 nm). (Figure 2C). X-ray diffraction (XRD) was employed to characterize the structural evolution of the LnNPs and LnNP@ZIF8-DOX (Figure 2D and Figure S4).

### 3.2. Response of LnNP to ROS

**Figure 3:**
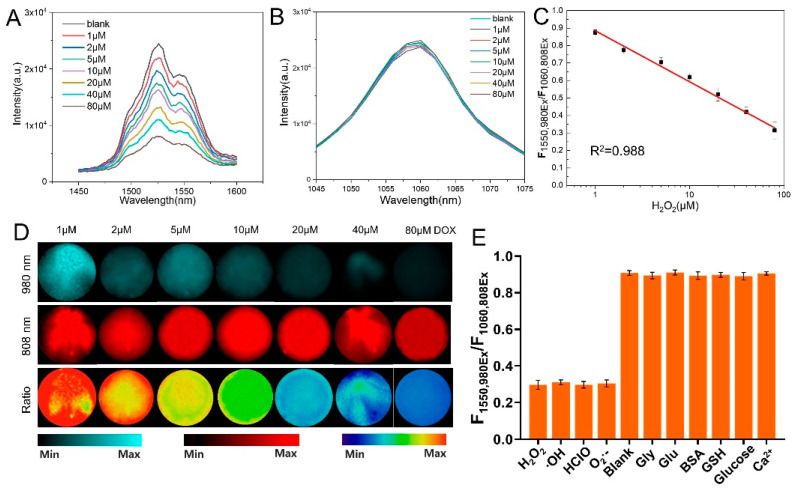
(**A**, **B**) NIR-II emission spectra of ligand-free LnNPs after the addition of different concentrations of H_2_O_2_ upon excitation at 980 nm (**A**) or 808 nm (**B**). (**C**) Calibration curve for the H_2_O_2_ assay. The linear relationship between F1550,980Ex/F1060,808Ex and H_2_O_2_ concentration in the range of 1 to 80 μM. (**D**) NIR-II imaging of the nanoprobe after being treated with different concentrations of GSH. Min: ratio = 0; Max: ratio = 1 (**E**) ratiometric F1550,980Ex/F1060,808Ex of the LnNPs responded to other ROS (80 μM) and various amino acids and ions (1 mM) in vitro. Error bars are obtained from three parallel samples. All experiments were performed independently at least three times.

Since Ce^3+^ ions in the host matrix were exposed on the surface of the ligand-free NPs after the acid treatment, H_2_O_2_ or other ROS species can directly oxidize Ce^3+^ to Ce^4+^ through redox reactions, resulting in the quenching of the NIR-II emission of Er^3+^ upon excitation at 980 nm [26,27]. Benefiting from such redox reactions, LnNPs can be explored as an effective probe for the detection of ROS. Next, to investigate the quenching effect of ROS on the NIR-II emission of the LnNPs, ta ligand-free probe (0.5 mg/mL) was applied to react with different concentrations of H_2_O_2_ in the range of 0–80 μM. After the reaction, NIR-II fluorescent images and the intensity of the nanoprobe were acquired upon 808 or 980 nm excitation, respectively. It was observed that the fluorescent signal at 1550 nm of the nanoprobe gradually decreased with increasing H_2_O_2_ concentration upon irradiation with a 980 nm laser, due to the redox reaction between the H_2_O_2_ and Ce^3+^ ions (Figure 3A). However, the intensity of the NIR-II fluorescent signal at 1060 nm irradiated with an 808 nm laser remained stable (Figure 3B). As a result, the nanoprobe exhibited a ratiometric response for H_2_O_2_ in the range of 1 to 80 μM (Figure 3C) and the limit of detection (LOD) of H_2_O_2_ (calculated according to the following formula: LOD = 3 SD/K (SD: standard deviation of the response, K: the slope of the standard curve)) was determined to be 0.190 μM. NIR-II fluorescent imaging of the solution also showed that the signal at 1550 nm gradually became darker with increasing ROS concentration under irradiation with a 980 nm laser, whereas the signal at 1060 nm was largely unchanged after irradiation with an 808 nm laser. The ratiometric NIR-II image showed gradual dimming with increasing ROS concentration (Figure 3D). The selectivity of this probe was also tested. In the presence of various ROS species, the fluorescent signal at 1550 nm excited by a 980 nm laser (F1550,980Ex) was quenched to the same level, while the ratio of signal (F1550,980Ex/F1060,808Ex) remains stable under the interference of other amino acids and ions (Figure 3E). This suggested that the LnNP@ZIF8 probe had adequate sensitivity and universality for ROS species.

### 3.3. Drug Releasing of LnNP@ZIF8-DOX and Cellular Uptake

**Figure 4:**
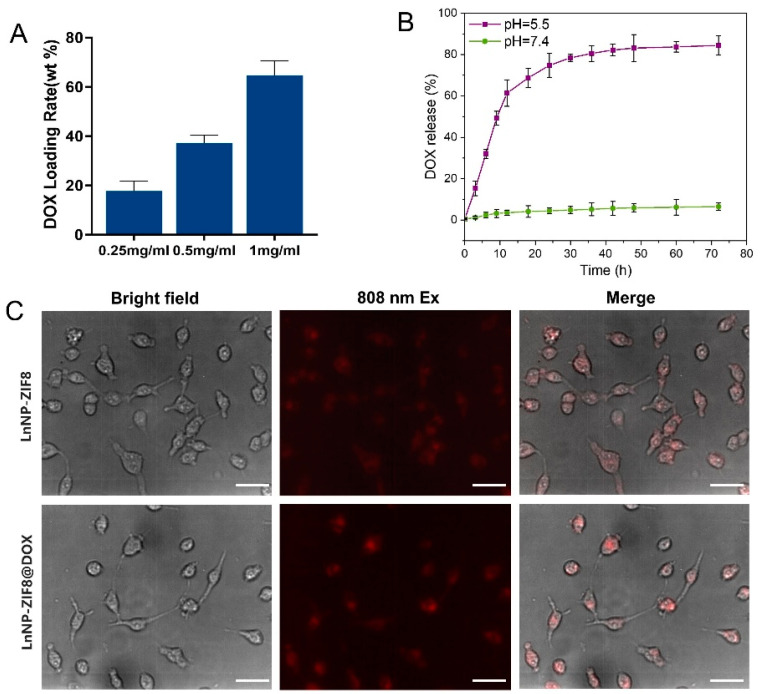
(**A**) Drug-loading capacity of NPs synthesized under 0.25 to 1 mg mL^−1^ DOX stock solution. (**B**) DOX release from LnNP@ZIF8-DOX immersed in bovine serum (pH ≈ 7.4) and acetate buffer solution (pH ≈ 5.5) for 72 h. Results are presented as means ± standard deviation (SD) (*n* = 3). (**C**) Confocal microscopic NIR images of MCF-7 cells treated with LnNP@ZIF8 and LnNP@ZIF8-DOX nanoprobes for 6 h (scale bar: 25 μm), λ_ex_ = 808 nm. All experiments were performed independently at least three times.

Firstly, we examined the drug-loading ability of freshly synthesized LnNP@ZIF8-DOX NPs. Different concentration of DOX stock solution was mixed with LnNP–PVP (5mg/mL), during the final step of the synthesis. The drug loading percentages of the nanoparticles were 17.8 wt%, 37.3 wt%, and 64.7 wt%, respectively (Figure 4A), determined by the UV/Vis analysis of unloaded drug in the supernatant following centrifuge treatment.

The successful encapsulation of DOX motivated us to study the degradation behavior of LnNP@ZIF8-DOX further. Owing to its acid instability, ZIF8 can be degraded under acid conditions, resulting in the release of embedded DOX. The responsive experiment under different pH conditions was carried out to confirm this specific degradation of ZIF8. It can be seen from Figure 4B that the ZIF-shell of the nano-assemblies collapsed under acetate buffer solution at pH 5.5, with a rapid release in the first 24 h and over 80% release after 72 h, whereas no obvious changes in the release amount of DOX were observed in bovine serum solution at pH 7.4 under 37 °C (below 10% in 3 days). Subsequently, we studied the cellular uptake of our nanoparticles. The near-infrared microscope images (Figure 4C) showed that the cell uptake of LnNP@ZIF8 and LnNP@ZIF8-DOX was efficient, which led to efficient drug delivery in wildtype MCF-7 cells without using a toxic organic solvent in the cell culture.

The ability of LnNP@ZIF8 and LnNP@ZIF8-DOX against cancer cells was evaluated by measuring the cell viability reduction after introducing the NPs to MCF-7 WT cells. The experiments were carried out by CCK-8 assay. The result showed that LnNP@ZIF8-DOX has a significant cytotoxic effect on MCF-7 wildtype cells, whereas LnNP@ZIF8 without DOX did not affect cell survival rate (Figure S5).

### 3.4. ROS Generation and Detection of LnNP@ZIF8-DOX In Vitro and In Vivo

**Figure 5:**
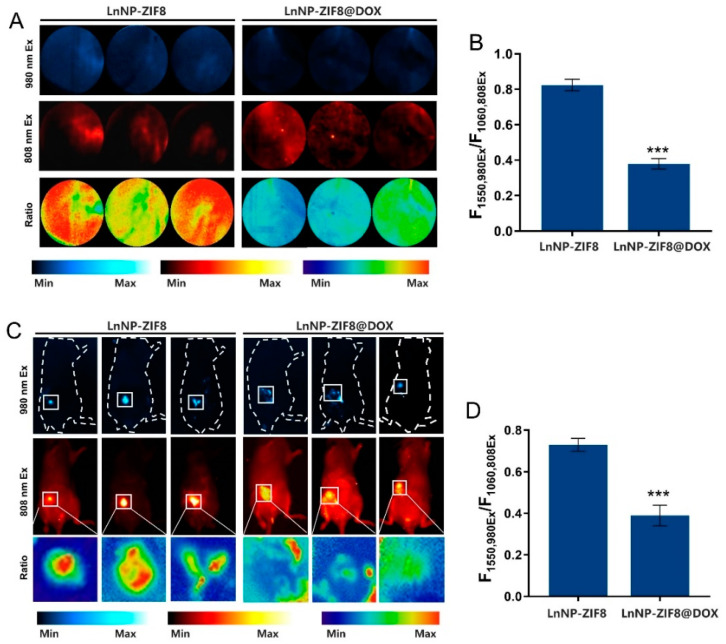
(**A**) NIR-II FL imaging of MCF-7 cells after incubation with LnNP@ZIF8 and LnNP@ZIF8-DOX, respectively. Min: ratio = 0; Max: ratio = 1. (**B**) The corresponding ratiometric F1550,980Ex/F1060,808Ex value of (**A**). (**C**) NIR-II imaging of 4T1 orthotopic tumor mice treated with LnNP@ZIF8 and LnNP@ZIF8-DOX under 808 and 980 nm laser irradiation and high magnification NIR-II ratio diagrams of tumor sites treated with nanoprobe. (**D**) The corresponding ratiometric F1550,980Ex/F1060,808Ex value of the tumor site ratio diagrams. All experiments were performed independently at least three times. *** *p* < 0.001 were determined by one-way ANOVA with Student’s *t* tests (two-tailed).

The main anticancer action of DOX is believed to be due to topoisomerase II inhibition and ROS generation [22]. Next, we tried to explore whether the nanoprobe could be applied to detect changes in ROS during LnNP@ZIF8-DOX co-culture with breast cancer cells. The MCF-7 cell line incubated in 12-well plates was divided into two groups for the following experiments. In the first group, MCF-7 cells were incubated with LnNP@ZIF8, which encapsulated no drugs, whereas cells of the other group were co-cultured with LnNP@ZIF8-DOX. After incubation for 24 h, NIR-II images irradiated with 808 or 980 nm laser light were recorded. The results showed that the cells treated with LnNP@ZIF8 alone were not able to quench the NIR-II emission of nanoprobes upon 980 nm excitation, since no ROS was generated. In the second group, MCF-7 cells treated with LnNP@ZIF8-DOX for 24 h showed relatively weak F1550,980Ex signals, and weak ratiometric signals were observed, indicating that some extent of ROS was generated in cells by DOX released from the nano-assemblies (Figure 5A). In the first group, the ratio was 0.824 ± 0.026, and ROS mean concentration in the MCF-7 cells was 1.618 μM compared with the standard curve. In the second group, the ratio was 0.406 ± 0.065, and the calculated ROS mean concentration in the MCF-7 cells was 43.787 μM (Figure 5B). These data illustrated the feasibility of the ratiometric nanoprobe for intracellular ROS detection.

Given the excellent performance of the nanoprobe for the generation and detection of ROS in vitro, we further explored its applicability for ROS detection in vivo by using the 4T1 orthotopic breast cancer mouse model. The nanoprobes were injected into the 4T1 tumors. After 24 h, NIR-II imaging of the whole mice was recorded with 808 or 980 nm laser light. Consistent with the in vitro results, the orthotopic tumors treated with LnNP@ZIF8-DOX emitted relatively weaker fluorescence at 1550 nm irradiated with a 980 nm laser compared with tumors treated with LnNP@ZIF8, while emitting stable fluorescence at 1060 nm irradiated with a 980 nm laser (Figure 5C). In addition, uptake, and distribution of DOX in tumor tissues after the injection of LnNP@ZIF8-DOX were shown by immunofluorescence images (Figure S6). The fluorescence changes of the nanoprobes in tumors over time were studied (Figure S7). At the same time, nanoprobe safety was also demonstrated by H&E staining (Figure S8). Based on the requirement for timeliness and comparative imaging results, subsequent studies using 24 h imaging are discussed. In the LnNP@ZIF8 group, the ratio at 24 h was 0.729 ± 0.025, and the tested mean ROS level (converted to H_2_O_2_) in 4T1 tumors of mice was 3.426 μM compared with the standard curve (Figure 5D). In the LnNP@ZIF8-DOX group, the ratio was 0.389 ± 0.041, and the calculated mean ROS concentration (converted to H_2_O_2_) in 4T1 tumor tissues was 50.061 μM (Figure 5D). This result further proved that the nanoprobe could accurately quantify ROS in mice and possess excellent performance in tumor imaging.

### 3.5. Capability of Identifying DOX Resistance

**Figure 6:**
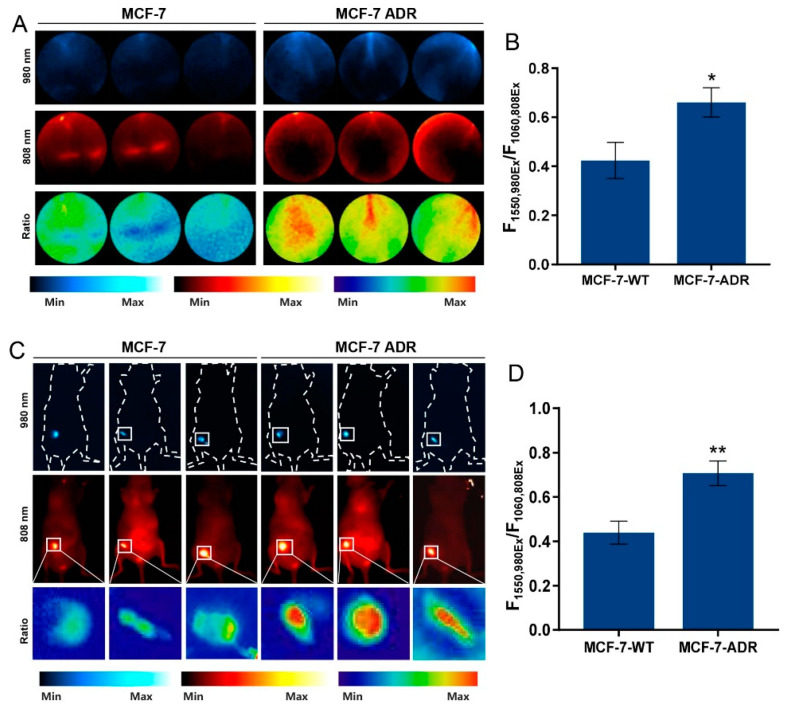
(**A**) NIR-II imaging of MCF-7-WT and MCF-7-ADR cells after incubation with LnNP@ZIF8-DOX. Min: ratio = 0; Max: ratio = 1. (**B**) The corresponding ratiometric F1550,980Ex/F1060,808Ex value of (**A**). (**C**) NIR-II imaging of MCF-7-WT and MCF-7-ADR orthotopic tumor mice treated with LnNP@ZIF8-DOX under 808 and 980 nm laser irradiation and high magnification NIR-II ratio diagrams of tumor sites treated with nanoprobes. (**D**) The corresponding ratiometric F1550,980Ex/F1060,808Ex value of the tumor site ratio diagrams. All experiments were performed independently at least three times. * *p* < 0.05 and ** *p* < 0.01, were determined by one-way ANOVA with Student’s *t* tests (two-tailed).

ROS generated by DOX might exceed a certain ROS threshold in DOX-sensitive cells, resulting in detrimental oxidative stress and ultimately apoptosis or necrosis. In contrast, DOX-resistant cells are able to counteract increased ROS levels through their endogenous antioxidant systems. Thus, monitoring the ROS level is a hopeful strategy for predicting doxorubicin resistance, which inspired us to apply our ROS-sensing probe to identifying DOX resistance [28]. Experiments were carried out on wildtype MCF-7 (MCF-7-WT) and MCF-7-ADR, a breast cancer cell line and its DOX-resistant counterpart, to explore the nanoprobe’s identification ability. Cells were incubated with LnNP@ZIF8-DOX for 24 h, and then NIR-II images were recorded. As we can see from Figure 6A, the emission at 1550 nm irradiated with a 980 nm laser was distinctly quenched by ROS generated by LnNP@ZIF8-DOX, which is consistent with the results given above. It is noteworthy that, in MCF-7-ADR cells, emission at 1550 nm irradiated with a 980 nm laser maintains a certain intensity, and the ratio diagrams replaced with the pseudo-colored version still showed brightly. The calculated NIR-II FL ratio of MCF-7-WT and MCF-7-ADR cells at 24 h was 0.424 ± 0.060 and 0.661 ± 0.049, respectively (Figure 6B). Compared with the standard curve, the mean level of ROS (converted to H_2_O_2_) was only 5.905 μM, while the concentration in the MCF-7-WT group was 37.985 μM (Figure 6B). To confirm that DOX kept its therapeutic potential and the MCF-7-ADR cells acquired DOX resistance, we explored the IC_50_ of LnNP@ZIF8-DOX for the MCF-7-WT cells and MCF-7-ADR cells (Figure S9). Furthermore, we tested the nanoprobe’s ability to identify DOX resistance in vivo. In this study, we developed a human breast tissue-derived orthotopic mouse model of breast cancer, in which the normal human breast tissues were implanted subcutaneously to create a normal human mammary microenvironment, after which MCF-7-WT and MCF-7-ADR cells were inoculated into the implants [29]. After 24 h of intratumor injection of the probe, a significantly decreased signal at 1550 nm irradiated with a 980 nm laser was observed in the MCF-7-WT cells, indicating an intense response of nanoprobes to ROS (Figure 6C, left). However, the MCF-7-ADR tumor tissues treated with LnNP@ZIF8-DOX emitted obvious NIR-II signals in both irradiation wavelengths and showed a bright ratiometric signal (Figure 6C, right). According to the data of the MCF-7-WT group, the NIR-II fluorescence ratio of MCF-7-WT and MCF-7-ADR tissues at 24 h was 0.440 ± 0.042 and 0.708 ±0.045, respectively (Figure 6D). Based on the standard curve, the mean ROS concentration (converted to H_2_O_2_) of MCF-7-WT tumor tissues in situ was 33.481 μM, while the level in the MCF-7-ADR group was 4.075 μM (Figure 3C and Figure 6D).

Here, these data show that LnNP@ZIF8-DOX is uniquely sensitive to the upregulated antioxidant pathways present in drug-resistant human breast tumors. In the drug-resistant cell lines and tumor tissues, lower ratio intensity corresponded with decreased ROS concentrations in comparison to drug-sensitive tumors. Therefore, our redox imaging agent enables the identification of patients with breast cancer that are refractory to the standard of care, as well as monitoring their response posttreatment.

## 4. Conclusions

In conjunction with DNA damage, chemotherapies generate high levels of oxidative stress in tumors and lead to the apoptosis of cells sensitive to treatment [30]. However, drug resistance has become a major obstacle to the clinical use of doxorubicin [31,32]. In addition, there is no satisfactory way to identify patients who become refractory to the standard of care. DOX-resistant cancer cells can maintain redox homeostasis to protect themselves from the harmful effects of oxidative stress, thereby conferring treatment resistance. Consequently, elevated antioxidant capacity is an ideal hallmark of doxorubicin insensitivity in breast cancer cells [33]. ROS-sensing probes have the potential to assess the efficacy of chemotherapeutics that converge with the induction of oxidative stress. In a recent study, the ^18^F-FSPG imaging system, reflecting the tumor antioxidant response to doxorubicin, was shown to be a promising biomarker of drug resistance in animal models of ovarian cancer [28]. Among the optical sensing methods currently being explored, ratiometric fluorescence sensing has received particular attention as a technique with the potential to provide precise and quantitative analyses [34,35,36].

In our study, the acidic tumor microenvironment was exploited to respond to pH-sensitive doxorubicin-loaded LnNP@ZIF8, resulting in the rapid release of doxorubicin at low pH and an increase in ROS levels. Furthermore, the core-shell LnNPs showed ultrasensitive performance for ROS detection. The findings demonstrate that ZIFs are prime candidates as drug carriers for monitoring drug efficacy. Carrying out matched drug-sensitive and drug-resistant breast cancer cells, we confirmed that resistance to doxorubicin corresponded with an increase in tumor antioxidant capacity, defined through the decrease in ROS [37]. lower ROS corresponded to a decreased emission signal at 1550 nm in drug-resistant versus drug-sensitive cells.

Together, we envisage this nano-assembled platform has the potential to predict drug resistance prior to invalid chemotherapy. Ultimately, this would result in fewer ineffective cures and a better prognosis.

## Data Availability

Not applicable.

## Supplementary Materials

The following supporting information can be downloaded at: https://www.mdpi.com/article/10.3390/nano12244478/s1, Figure S1: NIR-II emission spectra of LnNP treated with the addition of ROS or not upon excitation at 808 nm; Figure S2: NIR-II emission spectra of LnNP treated with the addition of ROS or not upon excitation at 980 nm; Figure S3: TEM images of core of LnNP core, LnNP CS1 and LnNP CS1S2; Figure S4: XRD patterns of LnNP-OA; Figure S5: cell viability of MCF-7-WT cells with different treatments; Figure S6: uptake and distribution of DOX in tumor tissues; Figure S7. NIR-II FL imaging of MCF-7 tumor mice at different time points; Figure S8. H&E staining images of Vital organs. Figure S9. IC50 of the LnNP@ZIF8-DOX in the MCF-7-WT and MCF-7-ADR cells.

## References

[1] Oner G., Altintas S., Canturk Z., Tjalma W., Verhoeven Y., Van Berckelaer C., Berneman Z., Peeters M., Pauwels P., Dam P.A. (2019). Triple-Negative Breast Cancer-Role of Immunology: A Systemic Review. Breast J..

[2] Krasniqi E., Barchiesi G., Pizzuti L., Mazzotta M., Venuti A., Maugeri-Sacca M., Sanguineti G., Massimiani G., Sergi D., Carpano S. (2019). Immunotherapy in HER2-Positive Breast Cancer: State of the Art and Future Perspectives. J. Hematol. Oncol..

[3] Nicolini A., Ferrari P., Carpi A. (2022). Immune Checkpoint Inhibitors and Other Immune Therapies in Breast Cancer: A New Paradigm for Prolonged Adjuvant Immunotherapy. Biomedicines.

[4] Twelves C., Jove M., Gombos A., Awada A. (2016). Cytotoxic Chemotherapy: Still the Mainstay of Clinical Practice for All Subtypes Metastatic Breast Cancer. Crit. Rev. Oncol. Hemat..

[5] Early Breast Cancer Trialists’ Collaborative Group (EBCTCG) (2005). Effects of Chemotherapy and Hormonal Therapy for Early Breast Cancer on Recurrence and 15-Year Survival: An Overview of the Randomised Trials. Lancet.

[6] Untch M., Jackisch C., Schneeweiss A., Schmatloch S., Aktas B., Denkert C., Schem C., Wiebringhaus H., Kümmel S., Warm M. (2019). NAB-Paclitaxel Improves Disease-Free Survival in Early Breast Cancer: GBG 69-GeparSepto. J. Clin. Oncol..

[7] Schmid P., Adams S., Rugo H.S., Schneeweiss A., Barrios C.H., Iwata H., Dieras V., Hegg R., Im S.A., Shaw Wright G. (2018). Atezolizumab and Nab-Paclitaxel in Advanced Triple-Negative Breast Cancer. N. Engl. J. Med..

[8] Pilco-Ferreto N., Calaf G.M. (2016). Influence of Doxorubicin on Apoptosis and Oxidative Stress in Breast Cancer Cell Lines. Int. J. Oncol..

[9] Traverso N., Ricciarelli R., Nitti M., Marengo B., Furfaro A., Pronzato M., Marinari U., Domenicotti C. (2013). Role of Glutathione in Cancer Progression and Chemoresistance. Oxid. Med. Cell. Longev..

[10] Chen Y., Li Y., Huang L., Du Y., Gan F., Li Y., Yao Y. (2021). Antioxidative Stress: Inhibiting Reactive Oxygen Species Production as a Cause of Radioresistance and Chemoresistance. Oxid. Med. Cell. Longev..

[11] Singer E., Judkins J., Salomonis N., Matlaf L., Soteropoulos P., McAllister S., Soroceanu L. (2015). Reactive Oxygen Species-Mediated Therapeutic Response and Resistance in Glioblastoma. Cell Death Dis..

[12] Yamamoto M., Kensler T.W., Motohashi H. (2018). The KEAP1-NRF2 System: A Thiol-Based Sensor-Effector Apparatus for Maintaining Redox Homeostasis. Physiol. Rev..

[13] Kennedy L., Sandhu J., Harper M., Cuperlovic-Culf M. (2020). Role of Glutathione in Cancer: From Mechanisms to Therapies. Biomolecules.

[14] Hong G., Antaris A.L., Dai H. (2017). Near-Infrared Fluorophores for Biomedical Imaging. Nat. Biomed. Eng..

[15] Zhu S., Tian R., Antaris A.L., Chen X., Dai H. (2019). Near-Infrared-II Molecular Dyes for Cancer Imaging and Surgery. Adv. Mater..

[16] Wang F., Zhu J., Wang Y., Li J. (2022). Recent Advances in Engineering Nanomedicines for Second Near-Infrared Photothermal-Combinational Immunotherapy. Nanomaterials.

[17] Fan Y., Liu L., Zhang F. (2019). Exploiting Lanthanide-Doped Upconversion Nanoparticles with Core/Shell Structures. Nano Today.

[18] Li D., He S., Wu Y., Liu J., Liu Q., Chang B., Zhang Q., Xiang Z., Yuan Y., Jian C. (2019). Excretable Lanthanide Nanoparticle for Biomedical Imaging and Surgical Navigation in the Second Near-Infrared Window. Adv. Sci..

[19] Lu K., Aung T., Guo N., Weichselbaum R., Lin W. (2018). Nanoscale Metal-Organic Frameworks for Therapeutic, Imaging, and Sensing Applications. Adv. Mater..

[20] Hao C., Wu X., Sun M., Zhang H., Yuan A., Xu L., Xu C., Kuang H. (2019). Chiral Core-Shell Upconversion Nanoparticle@MOF Nanoassemblies for Quantification and Bioimaging of Reactive Oxygen Species In Vivo. J. Am. Chem. Soc..

[21] Li B., Tian F., Cui X., Xiang B., Zhao H., Zhang H., Wang D., Li J., Wang X., Fang X. (2022). Review for Rare-Earth-Modified Perovskite Materials and Optoelectronic Applications. Nanomaterials.

[22] Mizutani H., Tada-Oikawa S., Hiraku Y., Kojima M., Kawanishi S. (2005). Mechanism of Apoptosis Induced by Doxorubicin through the Generation of Hydrogen Peroxide. Life Sci..

[23] Lennicke C., Cochemé H. (2021). Redox Metabolism: ROS as Specific Molecular Regulators of Cell Signaling and Function. Mol. Cell.

[24] Gnanasammandhan M., Idris N., Bansal A., Huang K., Zhang Y. (2016). Near-IR Photoactivation Using Mesoporous Silica-Coated NaYF_4_:Yb,Er/Tm Upconversion Nanoparticles. Nat. Protoc..

[25] Zhang X., Chen W., Xie X., Li Y., Chen D., Chao Z., Liu C., Ma H., Liu Y., Ju H. (2019). Boosting Luminance Energy Transfer Efficiency in Upconversion Nanoparticles with an Energy-Concentrating Zone. Angew. Chem. Int. Ed..

[26] Ho T.-H., Yang C.-H., Jiang Z.-E., Lin H.-Y., Chen Y.-F., Wang T.-L. (2022). NIR-Triggered Generation of Reactive Oxygen Species and Photodynamic Therapy Based on Mesoporous Silica-Coated LiYF4 Upconverting Nanoparticles. Int. J. Mol. Sci..

[27] Lei X., Li R., Tu D., Shang X., Liu Y., You W., Sun C., Zhang F., Chen X. (2018). Intense Near-infrared-II Luminescence from NaCeF_4_:Er/Yb Nanoprobes for In Vitro Bioassay and In Vivo Bioimaging. Chem. Sci..

[28] Greenwood H.E., McCormick P.N., Gendron T., Glaser M., Pereira R., Maddocks O.D.K., Sander K., Zhang T., Koglin N., Lythgoe M.F. (2019). Measurement of Tumor Antioxidant Capacity and Prediction of Chemotherapy Resistance in Preclinical Models of Ovarian Cancer by Positron Emission Tomography. Clin. Cancer Res..

[29] Wang J., Xia T.-S., Liu X.-A., Ding Q., Du Q., Yin H., Wang S. (2009). A novel orthotopic and metastatic mouse model of breast cancer in human mammary microenvironment. Breast Cancer Res. Treat..

[30] Yang H., Villani R., Wang H., Simpson M., Roberts M., Tang M., Liang X. (2018). The Role of Cellular Reactive Oxygen Species in Cancer Chemotherapy. J. Exp. Clin. Cancer Res..

[31] Al-Malky H., Al Harthi S., Osman A. (2020). Major Obstacles to Doxorubicin Therapy: Cardiotoxicity and Drug Resistance. J. Oncol. Pharm. Pract..

[32] Schlam I., Tarantino P., Tolaney S.M. (2022). Overcoming Resistance to HER2-Directed Therapies in Breast Cancer. Cancers.

[33] Kankala R.K., Tsai P.Y., Kuthati Y., Wei P.R., Liu C.L., Lee C.H. (2017). Overcoming Multidrug Resistance Through Co-Delivery of ROS-Generating Nano-Machinery in Cancer Therapeutics. J. Mater. Chem. B.

[34] Zhao Y., Ji T., Wang H., Li S., Zhao Y., Nie G. (2014). Self-assembled peptide nanoparticles as tumor microenvironment activatable probes for tumor targeting and imaging. J. Control. Release.

[35] Ren W., Han J., Pradhan T., Lim J., Lee J., Lee J., Kim J., Kim J. (2014). A fluorescent probe to detect thiol-containing amino acids in solid tumors. Biomaterials.

[36] Shi H., He X., Wang K., Wu X., Ye X., Guo Q., Tan W., Qing Z., Yang X., Zhou B. (2011). Activatable aptamer probe for contrast-enhanced in vivo cancer imaging based on cell membrane protein-triggered conformation alteration. Proc. Natl. Acad. Sci. USA.

[37] Camphausen K., Citrin D., Krishna M., Mitchell J. (2005). Implications for Tumor Control During Protection of Normal Tissues with Antioxidants. J. Clin. Oncol..

