# Supplementary material for: LnNP@ZIF8 Smart System for In Situ NIR-II Ratiometric Imaging-Based Tumor Drug Resistance Evaluation
Source: Nanomaterials (Basel). 2022 Dec 17;12(24):4478. doi: 10.3390/nano12244478 (PMC9782036; doi:10.3390/nano12244478)
Supplement: Supplementary file 1 [file nanomaterials-12-04478-s001.zip › nanomaterials-2024788-supplementary.pdf]

Supplementary Materials

LnNP@ZIF8 Smart System for In Situ NIR-II Ratiometric Imaging-Based Tumor Drug Resistance Evaluation

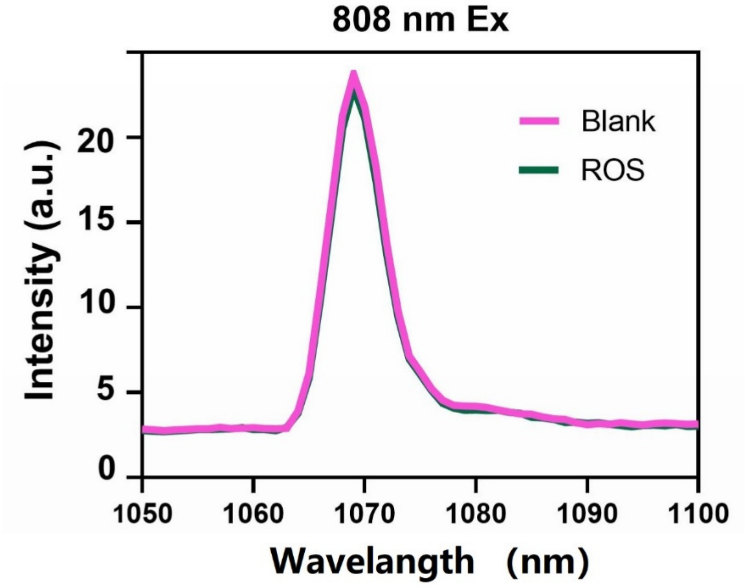

Figure S1. NIR-II emission spectra of LnNP treated with the addition of ROS or not upon excitation at 808 nm.

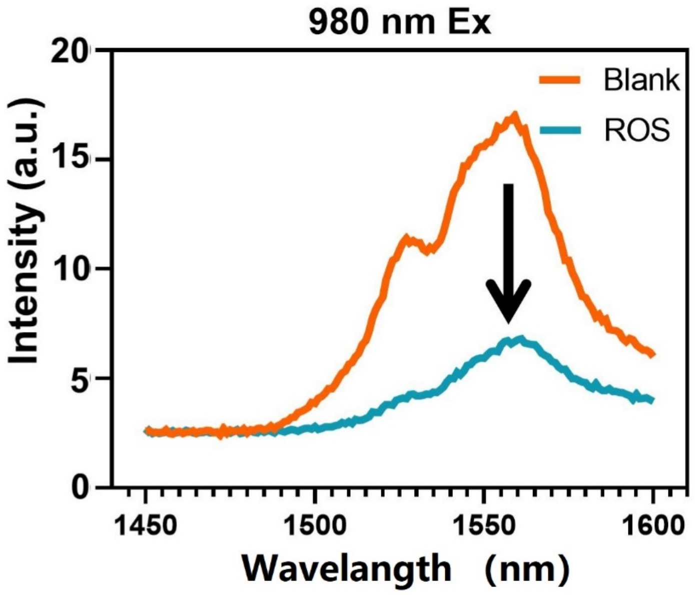

Figure S2. NIR-II emission spectra of LnNP treated with the addition of ROS or not upon excitation at 980 nm.

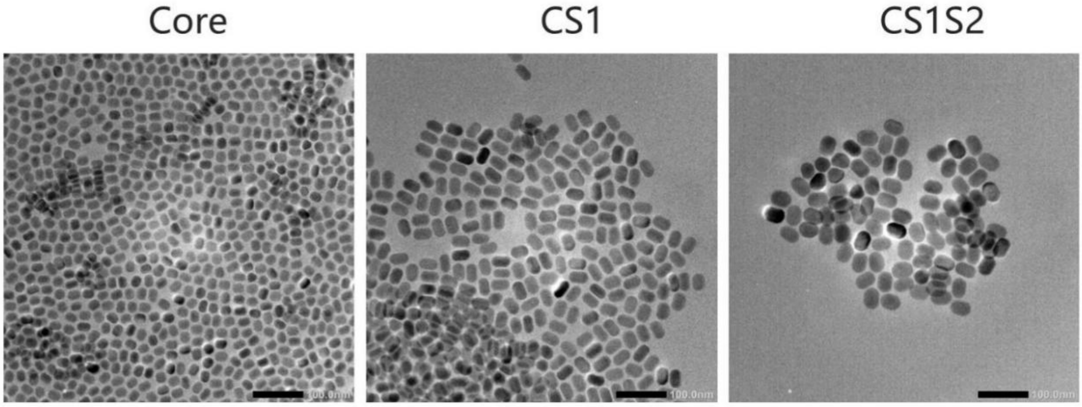

Figure S3. TEM images of core of LnNP core, LnNP CS1 and LnNP CS1S2 (Scale bar: 100 nm).

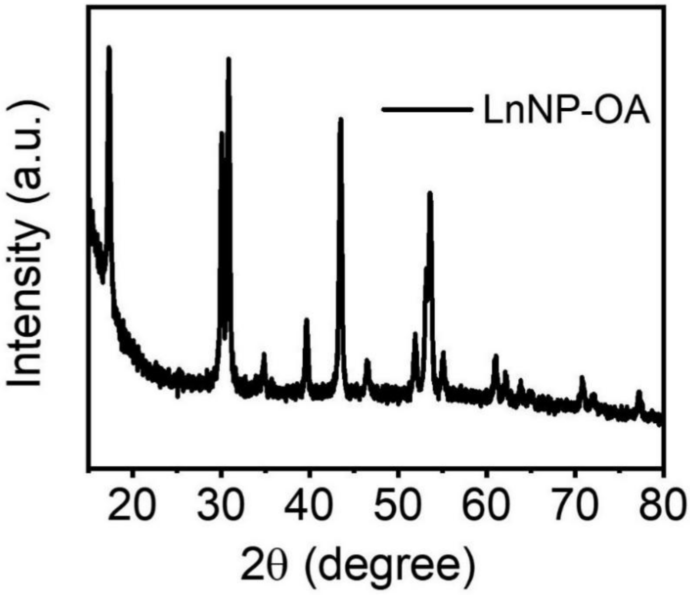

Figure S4. XRD patterns of LnNP-OA.

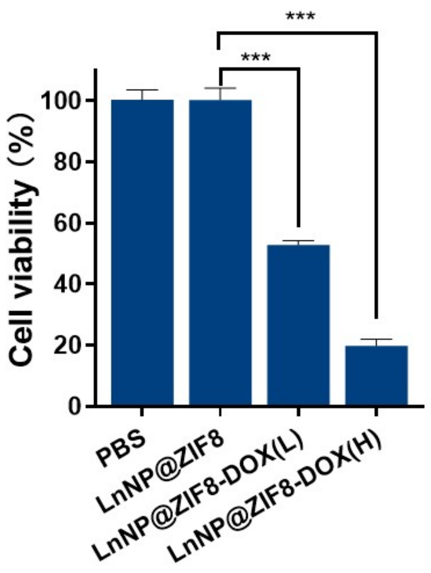

Figure S5. CCK-8 results of MCF-7-WT cells treated with PBS, LnNP@ZIF8, LnNP@ZIF8-DOX with lower DOX loading rate (LnNP@ZIF8-DOX(L)), and LnNP@ZIF8-DOX with higher DOX loading rate (LnNP@ZIF8-DOX(H)) after 24 h cultivation under equivalent DOX concentrations of 0, 0, 4.1, 17.0 μg/mL. Results are presented as means ± standard deviation (SD) (n=3, *p<0.05, **p<0.01, ***p<0.001).

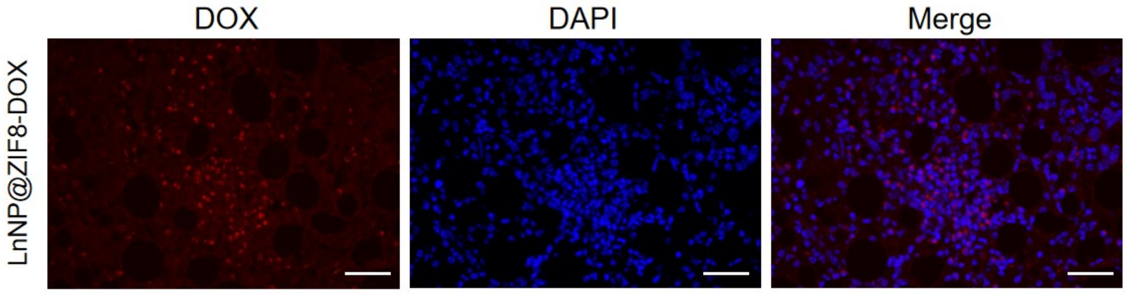

Figure S6. uptake and distribution of DOX in tumor tissues after the injection of LnNP@ZIF8-DOX (n = 3). Red: DOX; blue: DAPI. Scale bar: 100 μm.

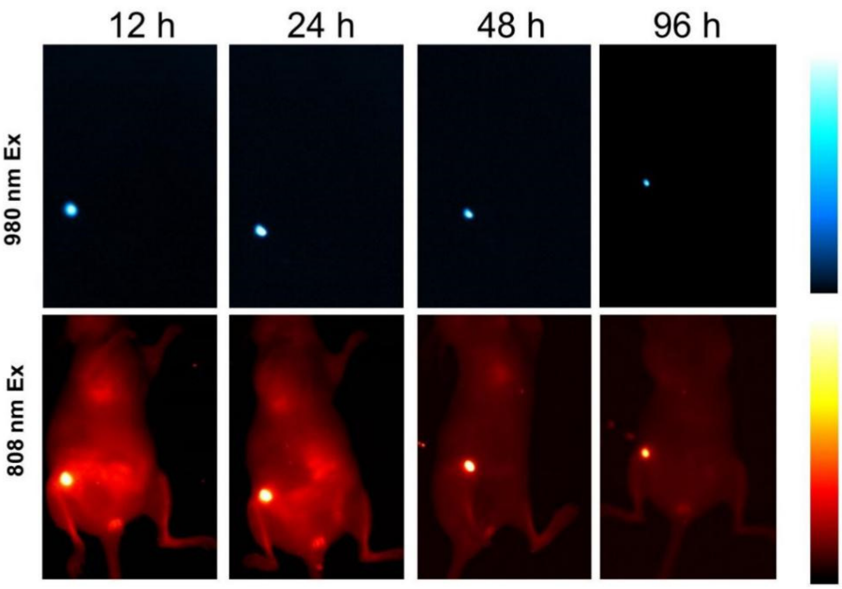

Figure S7. NIR-II FL imaging of MCF-7 tumor mice treated with LnNP-ZIF8@DOX under 808 and 980 nm laser irradiation at different time points.

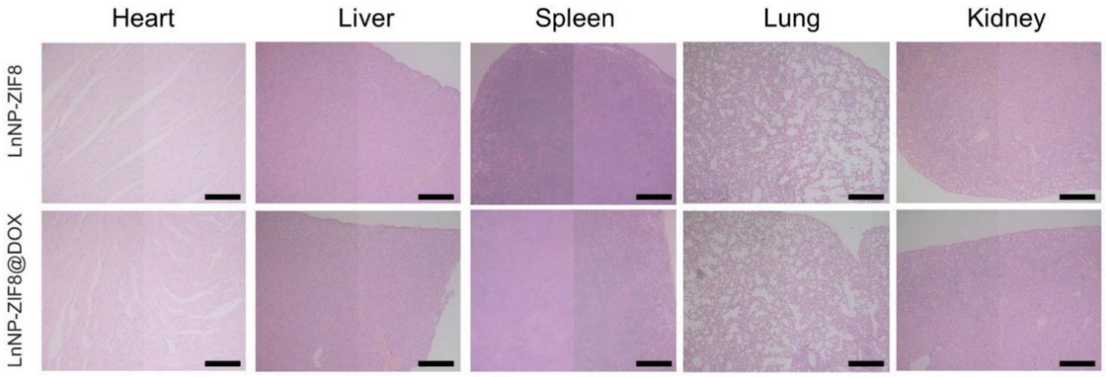

Figure S8. H&E staining images of heart, liver, spleen, lung and kidney in different experimental groups after 7 days of treatment. Scale bar: 200 μm.

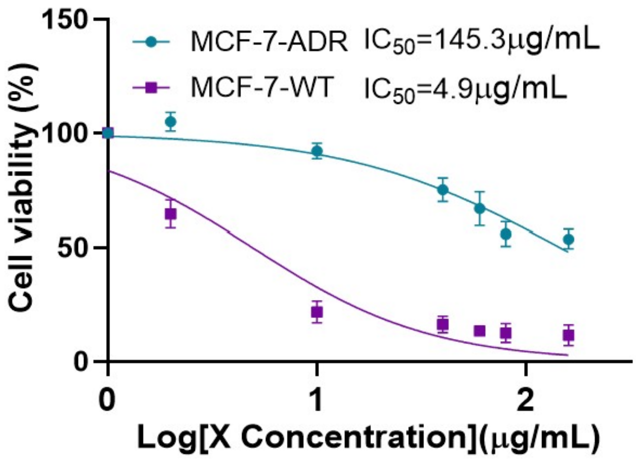

Figure S9. IC50 of the LnNP@ZIF8-DOX in the MCF-7-WT and MCF-7-ADR cells.X-axis: cell viability (%); y-axis: log (DOX concentration), (log (μg/mL)).
